# Single‐cell RNA sequencing reveals the multi‐cellular ecosystem in different radiological components of pulmonary part‐solid nodules

**DOI:** 10.1002/ctm2.723

**Published:** 2022-02-20

**Authors:** Yanmeng Li, Xiao Li, Haiming Chen, Kunkun Sun, Hao Li, Ying Zhou, Jun Wang, Fan Bai, Fan Yang

**Affiliations:** ^1^ Biomedical Pioneering Innovation Center (BIOPIC) School of Life Sciences & Department of Thoracic Surgery People's Hospital, Peking University Beijing China; ^2^ Department of Pathology Peking University People's Hospital Beijing China; ^3^ Beijing Advanced Innovation Center for Genomics (ICG) Peking University Beijing China

**Keywords:** early‐stage lung adenocarcinoma, part‐solid nodules, pulmonary subsolid nodules, single‐cell RNA sequencing, tumour microenvironment

## Abstract

**Background:**

Early‐stage lung adenocarcinoma that radiologically manifests as part‐solid nodules, consisting of both ground‐glass and solid components, has distinctive growth patterns and prognosis. The characteristics of the tumour microenvironment and transcriptional features of the malignant cells of different radiological phenotypes remain poorly understood.

**Methods:**

Twelve treatment‐naive patients with radiological part‐solid nodules were enrolled. After frozen pathology was confirmed as lung adenocarcinoma, two regions (ground‐glass and solid) from each of the 12 part‐solid nodules and 5 normal lung tissues from 5 of the12 patients were subjected to single‐cell sequencing by 10*x* Genomics. We used Seurat v3.1.5 for data integration and analysis.

**Results:**

We comprehensively dissected the multicellular ecosystem of the ground‐glass and solid components of part‐solid nodules at the single‐cell resolution. In tumours, these components had comparable proportions of malignant cells. However, the angiogenesis, epithelial‐to‐mesenchymal transition, KRAS, p53, and cell‐cycle signalling pathways were significantly up‐regulated in malignant cells within solid components compared to those within ground‐glass components. For the tumour microenvironment, the relative abundance of myeloid and NK cells tended to be higher in solid components than in ground‐glass components. Slight subtype composition differences existed between the ground‐glass and solid components. The T/NK cell subsets’ cytotoxic function and the macrophages’ pro‐inflammation function were suppressed in solid components. Moreover, pericytes in solid components had a stronger communication related to angiogenesis promotion with endothelial cells and tumour cells.

**Conclusion:**

The cellular landscape of ground‐glass components is significantly different from that of normal tissue and similar to that of solid components. However, transcriptional differences exist in the vital signalling pathways of malignant and immune cells within these components.

## Graphical abstract

- Single‐cell RNA sequencing reveals the multi‐cellular ecosystem in different radiological components of pulmonary part‐solid nodules.
- Pathways related to tumour cell proliferation, invasion, and aggression are significantly up‐regulated in malignant cells within solid components than those within ground‐glass components.
- Tumour microenvironment cells in solid components tend to show a stronger pro‐tumour function than those in ground‐glass components.

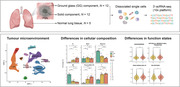

## 1 INTRODUCTION

Along with the widespread application of chest computed tomography (CT), the detection rate of early‐stage lung adenocarcinoma (LUAD) that radiologically manifests as subsolid nodules (SSNs) has increased considerably, particularly in never smokers.^1^, ^2^ Compared with solid nodules, SSNs have indolent growth patterns and excellent prognosis.^3^, ^4^ SSNs can be further categorized as pure ground‐glass (GG) nodules or part‐solid nodules (PSNs) according to the presence of solid components on thin‐section CT scans.^4^ Pathologically, radiological GG and solid opacities tend to correspond to lepidic (pre‐invasive) and invasive patterns, respectively.^4^ Clinically, for staging purposes, only the long‐axis dimension of the solid components within PSN is used. The size of the solid component is thought to be strongly correlated with the prognosis of the patients.^4^

Several studies have unravelled the evolutionary trajectory from pre‐invasive to invasive LUAD, but only a few of them have focused on SSNs.^5^, ^6^, ^7^, ^8^ Our previous study revealed branched evolution and remarkable genomic heterogeneity in SSNs.^9^ In addition to tumour cells, the tumour microenvironment (TME) plays a crucial role in shaping the biological and clinical behaviour of a tumour. Our team found that the ecosystem of SSNs is located at an interim between normal lung (nLung) tissue and solid LUAD.^10^ However, for PSNs consisting of two radiological components, whether the TME of GG components is similar to that of nLung tissues or invasive components and whether the TME undergoes a stepwise transition from GG to solid components remain undetermined.

In this study, we performed single‐cell RNA sequencing (scRNA‐seq) on 5 nLung tissues and 12 GG and 12 paired solid components of PSNs. We comprehensively characterized the ecosystems of GG components, solid components, and nLung tissues. We also identified differences in their cell‐type constitutions and molecular signature expressions. Our findings shed light on the underlying molecular characteristics of different radiological appearances and provide valuable biological insights into SSNs.

## 2 RESULTS

### 2.1 Single‐cell landscape of the nLung tissues and the GG and solid components of PSNs

**FIGURE 1:**
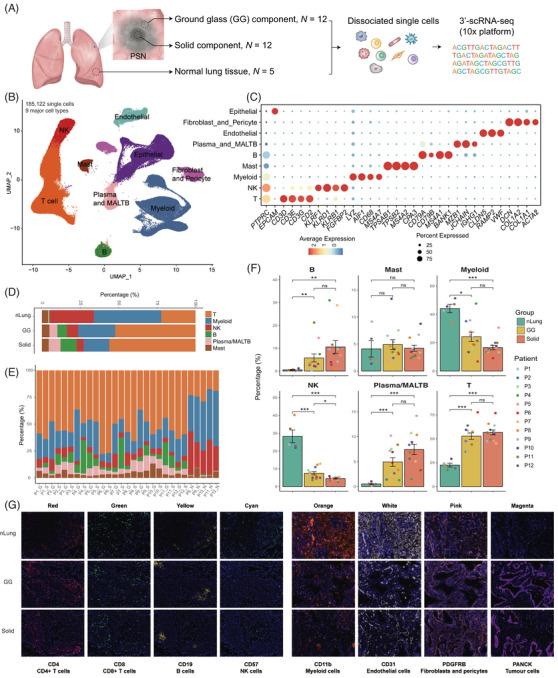
Study overview and dissection of the tumour microenvironment of the nLung tissues and the GG and solid components of PSNs using single‐cell RNA sequencing. (A) Schematic of the study design and sample information. (B) UMAP plot of 185,122 single cells coloured according to the nine major cell types. Cells were collected from nLung (*n* = 5), GG component (*n* = 12), and paired solid component (*n* = 12) samples. (C) Dot plot of expression of canonical marker genes for the nine major cell types. The dot size is proportional to the fraction of cells expressing the specific genes. The colour intensity corresponds to the relative expression of each specific gene. (D) Bar plot of the relative percentages of immune cell types across the three clinical groups. (E) Bar plot of the relative percentages of immune cell types across all samples. (F) Bar plots of the percentages of the six immune cell types among the three groups. *Y*‐axis: average percentage of samples across the three groups. Groups are shown in different colours. The error bars represent each group's ± standard error of the mean. The two‐sided unpaired Wilcoxon rank sum test was used for analysis. Multiple‐testing adjustment was performed using the Benjamini–Hochberg method. *P   < 0.05; **P   < 0.01; ***P   < 0.001. GG, ground‐glass; nLung, normal lung; ns: not significant; PSN, part‐solid nodule; UMAP, uniform manifold approximation and projection. (G) Immunohistochemistry staining showing the cellular components of nLung, GG, and solid components

We performed droplet‐based scRNA‐seq (10*x* Genomics) and collected 185122 high‐quality cell profiles from 12 treatment‐naive patients with PSNs (Figure 1A, Table S1). Together, 44777 cells were from the 5 nLung tissues, 70560 cells were from the 12 GG component samples, and 69785 cells were from the 12 paired solid component samples. All cells were catalogued into nine main clusters and annotated with canonical marker gene expression (Figure 1B,C, Table S2), including T cells, myeloid cells, natural killer (NK) cells, B cells, plasma and mucosa‐associated lymphoid tissue B cells (MALT B), mast cells, fibroblasts and pericytes, endothelial cells, and epithelial cells (alveolar and cancer cells).

To elucidate the immune cell dynamics in the nLung tissues and the GG and solid components, we compared the compositions of immune cell subsets after removing the epithelial and stromal populations in the three groups. The proportions in each clinical group and patient are shown in Figure 1D,E. The relative abundance of myeloid and NK cells was highest in the nLung tissues and tended to decline stepwise from the nLung tissues to the GG and solid component groups. Moreover, the relative proportions of T, B, plasma and MALT B cells tended to increase from GG components to solid components but without statistical significance. Their percentages in the GG and solid components were significantly higher than those in the nLung tissues, suggesting activation of adaptive immune responses in SSNs. Immunohistochemistry staining was further conducted to provide an overview of the multicellular ecosystems of nLung, GG and solid components (Figure 1G). These results suggested that both the GG and solid components of PSNs represent an active adaptive immune ecosystem when compared with nLung tissues, and slight differences in cell proportions were identified between the GG and solid components.

### 2.2 Intra‐tumour heterogeneity between GG and solid components

**FIGURE 2:**
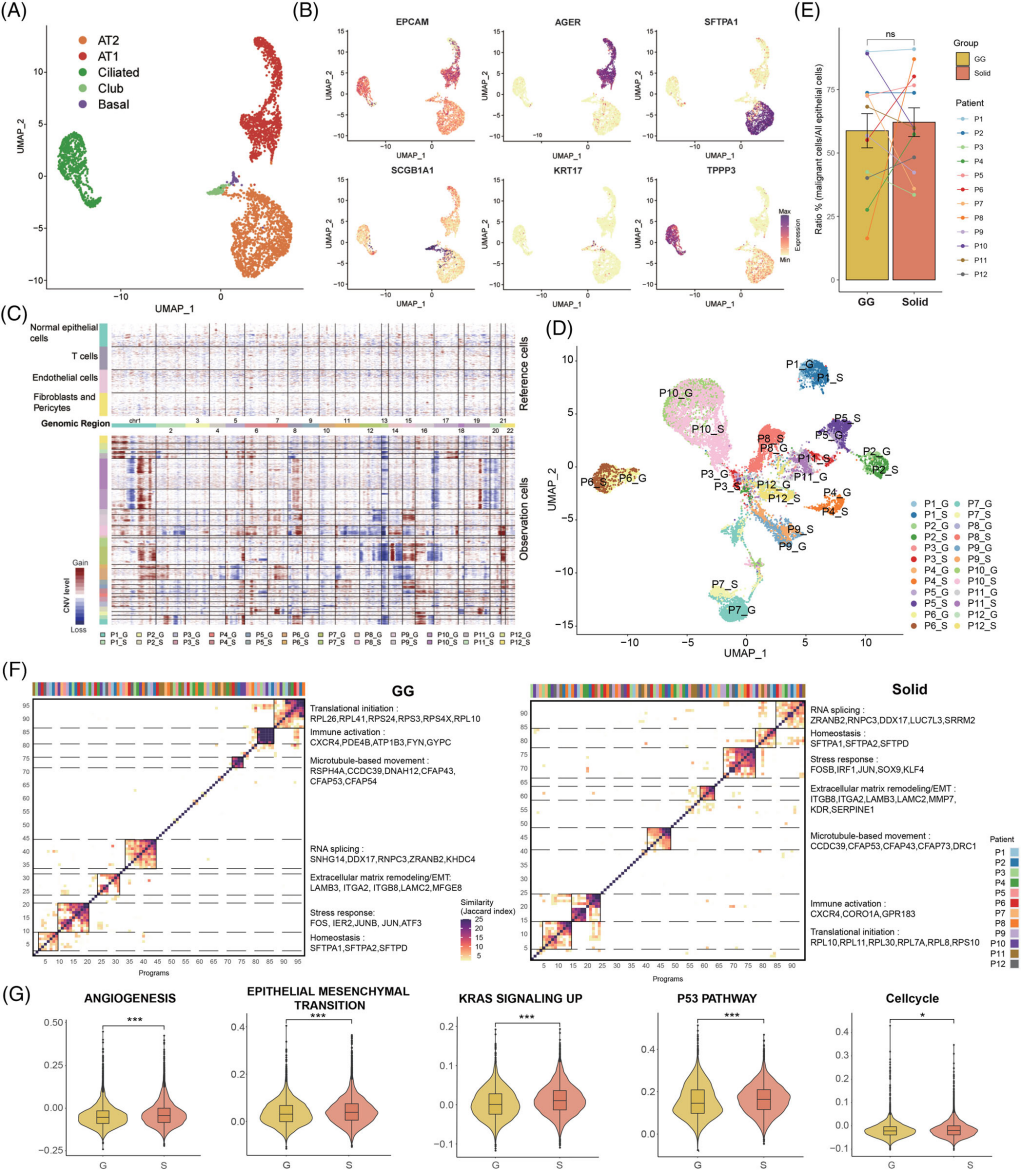
Identification and characterization of malignant cells in the GG and solid component samples. (A) Clustering of 3589 epithelial cells from nLung (*n* = 5) samples. Each dot corresponds to a single cell and is coloured according to cell type. (B) Feature plots of canonical markers, which were used to label the epithelial subtypes in the UMAP plots. (C) Heat map showing large‐scale CNVs that were inferred based on single‐cell RNA sequencing data of individual cells from GG and solid component samples. Non‐malignant cells were treated as references (top), and large‐scale CNVs were observed in malignant cells (bottom). The colour shows the log2 CNV ratio. Red: amplifications; blue: deletions. (D) UMAP plot of 16930 identified malignant cells from the GG and solid component samples. Each dot corresponds to a single cell and is coloured according to its origin. (E) Comparison of the proportions of malignant cells among all epithelial cells between the GG and solid component samples. The two‐sided paired Wilcoxon rank sum test was used for analysis. (F) The identified meta‐programs revealing the common features of the GG and solid components. Hierarchical clusters of pairwise similarities between non‐negative matrix factorization programs were identified across each GG and solid component sample. The functional annotations and the top genes are shown on the right. (G) Violin plots comparing the scores of the selected pathways between malignant cells from the GG and solid component groups. Linear mixed models with a random effect for patient were applied. *P*‐values of fixed effects (clinical group) were calculated using the Satterthwaite's method. *P   < 0.05; **P   < 0.01; ***P   < 0.001. CNV, copy number variation; GG, ground‐glass; nLung, normal lung; ns, not significant; UMAP, uniform manifold approximation and projection

Next, we characterized the transcriptional features of the main cell types. Within the epithelial cell cluster, 3589 cells were from normal tissues and were clustered as alveolar type I cells (AT1; AGER+), alveolar type II cells (AT2; SFTPA1+), secretory club cells (Club; SCGB1A1+), basal airway epithelial cells (Basal; KRT17+), and ciliated airway epithelial cells (Ciliated; TPPP3+) (Figure 2A,B). AT1 and AT2, which can initiate LUAD, were the most abundant subtypes.^11^ In tumour tissues, epithelial cells comprise both malignant cells and non‐malignant cells. We distinguished them by inferring large‐scale copy number variation (CNV) levels for each patient using the methods detailed in the Supporting information. Each malignant cell was identified with the CNV pattern having the Pearson correlation coefficient between its CNV pattern and the CNV reference vector (the calculated average CNV scores of the cells with a sum of CNV scores in the top 5%) above 0.3. Overall, 16930 malignant cells from 24 tumour samples were identified and retained for further tumour cell analyses.

At the genomic level, the malignant cells of the GG and solid components within one lesion shared similar CNV patterns (Figure 2C), suggesting that the genomic features were not correlated with the radiological features. Based on the transcriptional profiles, dimension reduction analyses of the tumour cells revealed patient‐specific clusters, highlighting the inter‐tumoural heterogeneity at the transcriptional level (Figure 2D). Moreover, the GG and solid samples had comparable proportions of malignant cells among all epithelial cells (Figure 2E), suggesting that the different radiological appearances were not determined by the degree of the normal‐to‐malignant transition.

To delineate the transcriptional features of cancer cells within GG and solid components and unravel their differences, we captured the common transcriptional patterns across lesions with the same radiological appearance. Non‐negative matrix factorization analysis was used to identify the full transcriptional spectrum of the intratumoural heterogeneity of each sample, as previously described.^12^ We performed hierarchical clustering to group the signatures of the lesions into main meta‐programs, revealing the common features of the GG and solid components that were independent of the patients. The highest concordance and the top co‐expressed genes among these programs within the two groups are shown in Figure 2F. The seven function modules, marked by the top‐scoring genes of the meta‐programs, were found to be identical in both clinical groups. They included the translational initiation program, immune activation program, microtubule‐based movement program, RNA splicing program, extracellular matrix remodelling/epithelial‐to‐mesenchymal transition (EMT) program, stress response program, and homeostasis program.

We next explored transcriptional differences between GG and solid components at the signalling pathway level by calculating the expression scores of the specific gene set pathways. The angiogenesis, EMT, KRAS signalling, p53 signalling, and cell‐cycle pathways were significantly up‐regulated in tumour cells within solid components compared to those within GG components (Figure 2G). These results suggested that the malignant cells of the two radiological phenotypes of SSNs had distinct functional patterns, which may cause differences in proliferation and invasiveness.

### 2.3 Suppressive cytotoxic function of T/NK cells in the solid components of SSNs

**FIGURE 3:**
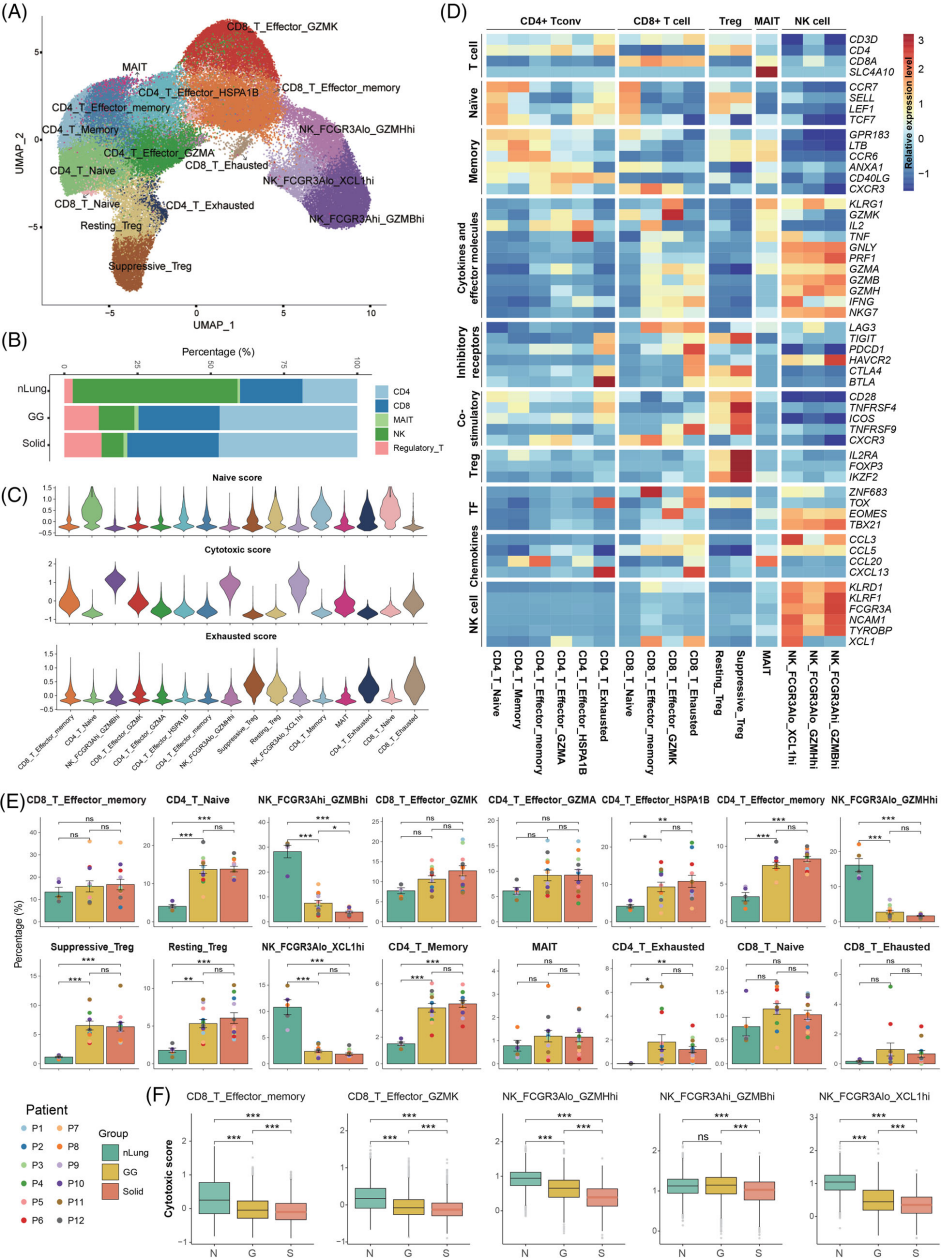
Composition and characterization of the T and NK subsets in the nLung tissues and the GG and solid components. (A) UMAP plot of 77562 T and NK cells revealing 16 subtypes. (B) Bar plot showing the relative percentages of CD4+, CD8+, MAIT, NK and Treg cells across the three clinical groups. (C) Violin plots showing the distribution of naive, cytotoxic and exhausted state scores in each T/NK cell subtype. (D) Heat map of the functional gene sets in the T and NK sub‐clusters. (E) Bar plots showing the average percentages of each T/NK cell subtype among the nLung tissues and the GG and solid component groups. Groups are shown in different colours. Error bars represent each group's ± standard error of the mean. The two‐sided unpaired Wilcoxon rank sum test was used for analysis. Multiple‐testing adjustment was performed using the Benjamini–Hochberg method. *P   < 0.05; **P   < 0.01; ***P   < 0.001. (F) Violin plots comparing the cytotoxic function scores between the 3 clinical groups in the selected subsets. Linear mixed models with a random effect for patient were applied. *P* values of fixed effects (clinical group) were calculated using the Satterthwaite's method. *P   < 0.05; **P   < 0.01; ***P   < 0.001. GG, ground‐glass; MAIT, mucosal‐associated invariant T cells; NK, natural killer; nLung, normal lung; ns, not significant; Treg, regulatory T cells; UMAP, uniform manifold approximation and projection

The sub‐clustering of 77562 T and NK cells revealed 16 subtypes clustered by heterogeneous cell lineages and functional states (Figure 3A): 6 subtypes of CD4+ T cells (CD3D+CD4+), 4 subtypes of CD8 T cells (CD3D+CD8A+), 3 subtypes of NK cells (CD3D‐CD56+FCGR3A+), 2 subtypes of regulatory T cells (Treg, CD4+FOXP3+TIGIT+), and mucosal‐associated invariant T cells (CD3D+SLC4A10+). Their relative percentages in the three groups and function scores are shown in Figure 3B,C.

For the CD4+ T cells, we identified naive (SELL+CCR7+), memory (CCR6+ LTB+), effector memory (CCR6+CCL20+), effector GZMA (GZMA+ GZMK+), effector HSPA1B (HSPA1B+IL2+), and exhausted (CXCL13+ ICA1+) CD4+ sub‐clusters (Figure 3A,D). As central players in the immune system, CD4+ T cells regulate both innate and adaptive immune responses.^13^ Overall, the relative percentages of these subtypes in the GG components were comparable to those in the solid components but were significantly higher than those in the nLung tissues (Figure 3E), suggesting the important role of CD4+ T‐mediated immunity in both of the radiological appearances of SSNs. Effector CD4+ T cells and effector memory CD4+ T cells were the prominent clusters in the tumours. We found that they were characterized by high expression of the cytokine IL2 (Figure 3D). IL2, originally called T cell growth factor, is produced primarily by activated T lymphocytes and increases the cell‐killing activity of both NK cells and cytotoxic T cells.^14^ Two effector CD4+ T cells, characterized by high expression of either IFNγ and TNF or GZMA and GZMK, showed an anti‐tumour function. In addition, exhausted CD4+ T cells, characterized by high expression of CXCL13 and BTLA, which are T follicular helper markers, were mainly collected from the tumour tissue.

For the CD8+ T cells, naive CD8+ T cells showed expression of SELL, CCR7, and LEF1 (Figure 3A,D). Memory CD8+ T cells were identified with ZNF683 and CXCR3 expression. GZMK+CD8+ effector T cells showed high expression of cytotoxic markers, such as GZMK, EOMES, and KLRG1. Exhausted CD8+ T cells were characterized by the low expression of cytotoxic markers, such as GZMK and NKG7, and high expression of inhibitory receptors and chemokines, such as LAG3, TIGIT, and BTLA. The CD8+ T subtypes had similar proportions in the three clinical groups (Figure 3E). However, the cytotoxic scores of CD8+ effector memory T cells and GZMK+CD8+ effector T cells were lowest in the solid component group, showing a weakened cytotoxic function compared with the GG component group (Figure 3F).

Both suppressive (CD4+FOXP3^high^IL2RA^high^) and resting (CD4+FOXP3^low^IL2RA^low^) Treg cells with high exhaustion scores expressed inhibitory receptors, such as CTLA4 and TIGIT. Treg cells were significantly enriched in the GG and solid components (Figure 3E), showing their immune‐suppressive role in SSNs.

Compared with the tumour samples, NK cells were significantly enriched in the nLung tissues. NK cells were sub‐clustered into three subsets, including CD16^high^GZMB^high^, CD16^low^GZMH^high^ and CD16^low^XCL1^high^ NK cells (Figure 3A). They highly expressed cytotoxic markers, such as PRF1, NKG7, and GNLY, and they presented the strongest cytotoxic function among all of the T and NK subtypes (Figure 3D). NK cells with high CD16 (FCGR3A) expression showed the strongest cytotoxic function (Figure 3C). The percentage of CD16^high^GZMB^high^ NK cells decreased stepwise from the nLung tissues to the GG and solid component groups. Moreover, the cytotoxic scores of the NK subtypes were significantly lower in the solid components than in the GG components (Figure 3F). We concluded that the immune response of NK cells was suppressed in the solid components when compared with the GG components and that this may promote immune escape in the more aggressive parts of PSNs.

### 2.4 Similar distribution of myeloid subtypes in the GG and solid components

**FIGURE 4:**
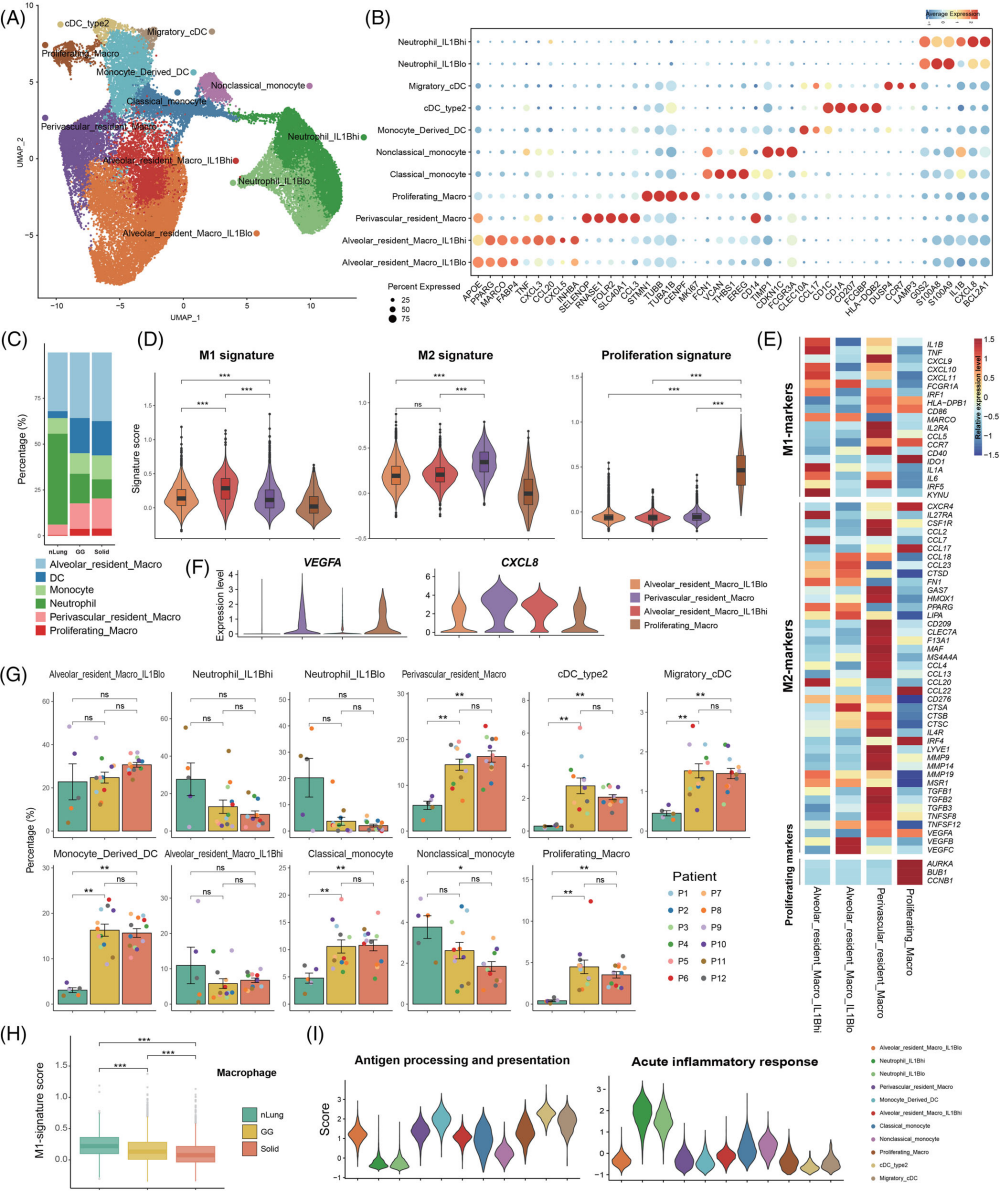
Composition and characterization of the myeloid subsets in the nLung tissues and the GG and solid components. (A) UMAP plot of 36110 myeloid cells revealing 11 subtypes. (B) Dot plot of the expression of canonical marker genes for each myeloid subset. The dot size is proportional to the fraction of cells expressing the specific genes. Colour intensity corresponds to the relative expression of the specific genes. (C) Bar plot showing the relative percentages of alveolar resident macrophages, DCs, monocytes, neutrophils, and proliferating macrophages across the three clinical groups. (D) Violin plots showing the distribution of M1 signature genes, M2 signature genes and proliferating state scores among each macrophage subtype. (E) Heat map of the functional gene sets in the macrophage subtypes. (F) Violin plots showing the expression of angiogenesis‐related genes in each macrophage subset. (G) Bar plots showing the average percentages of each myeloid subtype among the nLung tissues and the GG and solid component groups. Groups are shown in different colours. Error bars represent each group's ± standard error of the mean. The two‐sided unpaired Wilcoxon rank sum test was used for analysis. Multiple‐testing adjustment was performed using the Benjamini–Hochberg method. *P   < 0.05; **P   < 0.01; ***P   < 0.001. (H) Box plots comparing the M1 signature scores between the three clinical groups in the selected subsets. Linear mixed models with a random effect for patient were applied. *P*‐values of fixed effects (clinical group) were calculated using the Satterthwaite's method. *P   < 0.05; **P   < 0.01; ***P   < 0.001. (I) Violin plots showing the distribution of function scores for antigen processing and presentation and acute inflammatory response in each myeloid subtype. DC, dendritic cell; GG, ground‐glass; nLung, normal lung; ns, not significant; UMAP, uniform manifold approximation and projection

Monocytes, macrophages, neutrophils, and dendritic cells (DCs) were identified by the sub‐clustering of 36110 myeloid cells (Figure 4A–C). Monocytes were sub‐clustered into classical monocytes (FCN1+CD14+VCAN+) and non‐classical monocytes (FCN1+ CD16+CDKN1C+). Four subtypes of macrophages were revealed, including proliferating macrophages (STMN1+CENPF+MKI67+), perivascular resident macrophages (LYVE1+LILRB5+SELENOP+), IL1B^high^ alveolar resident macrophages (PPARG+ FABP4+MARCO+) with high expression of IL1B, and IL1B^low^ alveolar resident macrophages (PPARG+FABP4+MARCO+) with low expression of IL1B. Macrophages are usually classified into the canonical pro‐inflammatory M1 and anti‐inflammatory M2 classes.^15^, ^16^ IL1B^high^ alveolar resident macrophages exhibited a pro‐inflammatory M1‐dominant gene signature, with relatively high expression of IL1B, IL1A, tumour necrosis factor, and CXCL10‐11 (Figure 4D,E). Perivascular resident macrophages exhibited an anti‐inflammatory M2‐dominant gene signature (e.g., CD40, CSF1R, CCL2) but also expressed few M1 signature genes (Figure 4D,E). In addition, they had high expression of VEGFA and CXCL8, which are related to angiogenesis (Figure 4F). Perivascular resident macrophages were more enriched in the GG and solid component groups than in the nLung tissues (Figure 4G), suggesting that they played a role in tumour progression. Moreover, macrophages in the solid components tended to have less M1‐dominant gene signature expression than the GG components (Figure 4H), suggesting different inflammatory statuses of macrophages in the two radiological phenotypes.

Three DC subtypes were identified, including monocyte‐derived DCs (FCGR2B+CCL17+CLEC10A+), migratory conventional DCs (CCR7+LAMP3+CCL22+) and type 2 conventional DCs (CD1A+CD207+HLA‐DQB2+). In line with our findings (Figure 4I), DCs excel at antigen presentation and play a key role in the induction of anti‐tumour T cell immunity.^17^ Each subtype was significantly enriched in the SSNs compared to in the nLung tissues (Figure 4G). For neutrophils (G0S2+S100A8+S100A9+), the IL1B^high^ and IL1B^low^ subtypes were identified. They showed the strongest acute inflammatory function among all of the myeloid subtypes (Figure 4I). Neutrophils are the first line of defence in the innate immune system,^18^ and they can release huge amounts of bioactive IL‐1β to regulate the resolution of inflammation.^19^, ^20^ We found that the percentage of neutrophils was higher in the nLung tissues than in either component of the SSNs, suggesting that the adaptive immune response was activated in the tumour.

### 2.5 Diverse functions of stromal subtypes between the GG and solid components

**FIGURE 5:**
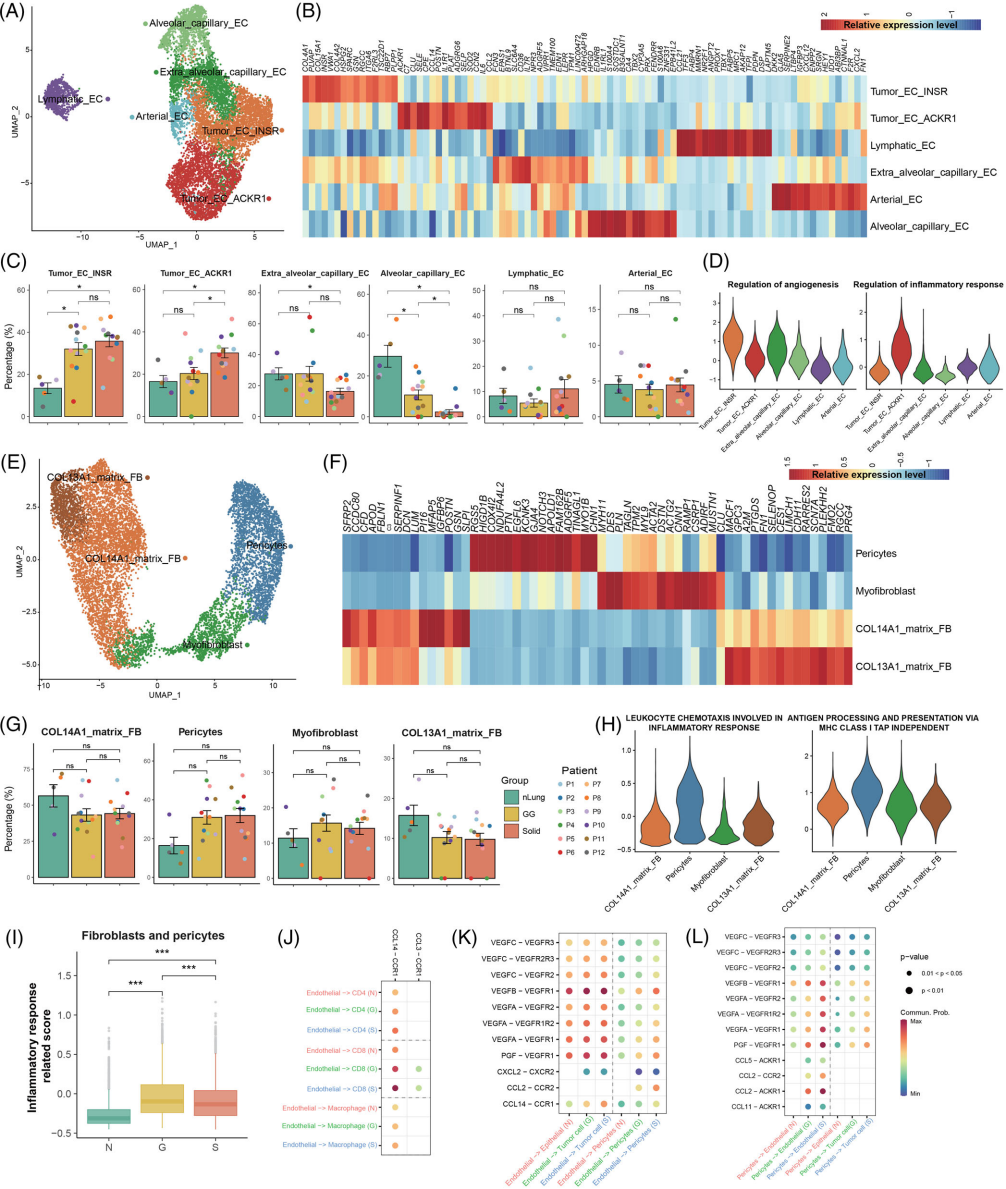
Composition and function of endothelial cell and fibroblast subsets in the nLung tissues and the GG and solid components. (A) UMAP plot of 10212 endothelial cells revealing six subtypes. (B) Heat map of the marker gene expression of the endothelial cell subsets. (C) Bar plots showing the average percentages of each endothelial subtype among the nLung tissues and the GG and solid component groups. Groups are shown in different colours. Error bars represent each group's ± standard error of the mean. The two‐sided unpaired Wilcoxon rank sum test was used for analysis. Multiple‐testing adjustment was performed using the Benjamini–Hochberg method. *P   < 0.05; **P   < 0.01; ***P   < 0.001. (D) Violin plots showing the distribution of function scores for regulation of angiogenesis and inflammatory response in each endothelial subtype. (E) UMAP plot of 10140 fibroblasts revealing four subtypes. (F) Heat map of the marker gene expression of the fibroblast subsets. (G) Bar plots showing the average percentages of each fibroblast subtype among the nLung tissues and the GG and solid component groups. Groups are shown in different colours. Error bars represent each group's ± standard error of the mean. The two‐sided unpaired Wilcoxon rank sum test was used for analysis. Multiple‐testing adjustment was performed using the Benjamini–Hochberg method. *P   < 0.05; **P   < 0.01; ***P   < 0.001. (H) Violin plots showing the distribution of function scores for inflammatory response and antigen processing and presentation in each fibroblast subtype. (I) Box plots comparing the inflammatory response scores between the three clinical groups in terms of fibroblasts and pericytes. Linear mixed models with a random effect for patient were applied. *P* values of fixed effects (clinical group) were calculated using the Satterthwaite's method. *P   < 0.05; **P   < 0.01; ***P   < 0.001. (J–L) Dot plots showing the cell–cell interactions of selected ligand–receptor interactions between different cell types in the three clinical groups. Dot colour reflects communication probability. Dot size represents computed *P*‐value, which was computed using the one‐sided permutation test. Empty spaces indicate that the communication probability was zero. GG, ground‐glass; nLung, normal lung; ns, not significant; UMAP, uniform manifold approximation and projection

To depict the stromal cell landscape of the nLung tissues and the different radiological components of SSNs, we obtained 10212 endothelial cells (ECs) and 10140 fibroblasts. The sub‐clustering of ECs revealed six subtypes, including extra‐alveolar capillary EC (cEC) (FCN3+EDN1+PLVAP+), alveolar cEC (HPGD+EDNRB+IL1RL1+), arterial EC(GJA5+FBLN5+DKK2), lymphatic EC (CCL21+TFF3+FABP4), INSR+ tumour EC (INSRhiHSPG2+PLVAP+), and ACKR1+ tumour EC (ACKR1+SELP+IL1R1+) (Figure 5A). The top expressed genes are shown in Figure 5B. The up‐regulated expression of INSR is a marker of tumour‐associated endothelial cells and is functionally involved in angiogenesis.^21^, ^22^, ^23^ We found that the INSR+ tumour endothelial cells were more enriched in the SSNs than in the normal tissues (Figure 5C), illustrating the remodelling of endothelial cell subtypes in the TME. Functionally, they demonstrated strong activation of angiogenesis regulation (Figure 5D). Compared with normal tissues and lesions with the GG radiological appearance, ACKR1+ tumour ECs were significantly enriched in lesions with the solid radiological appearance (Figure 5C). ACKR1 is expressed on the endothelial cells of tumour‐associated blood vessels.^24^ ACKR1+ tumour ECs showed that genes related to inflammatory response regulation were strongly up‐regulated (Figure 5D), suggesting strong immune activation in the solid components.

For fibroblasts, four known subtypes were identified: COL14A1+ (COL14A1+GSN+PI16+CFD+) and COL13A1+ (COL13A1+GPC3+NPNT+) matrix fibroblasts, myofibroblasts (ACTA2+MYH11+TAGLN+), and pericytes (RGS5+NOTCH3+HIGD1B+) (Figure 5E,F). The distribution of fibroblast subtypes in the GG components was similar to that in the solid components (Figure 5G). In contrast to normal tissues, pericytes in both the GG and solid components tended to be enriched but without statistical significance. Lung pericytes are located in the perivascular niche and are the progenitors of myofibroblasts, and they are capable of responding to danger signals and amplifying the inflammatory response through the elaboration of cytokines and adhesion molecules.^25^ We found that the pericytes in the up‐regulated gene sets of the SSNs were related to the leukocyte chemotaxis involved in the inflammatory response and in antigen presentation via MHC class I molecules, suggesting that they play a role in immune modulation in the TME (Figure 5H). Moreover, fibroblasts and pericytes in the GG components had higher immune response scores than those in the solid components (Figure 5I).

Stromal cells play vital roles in intercellular interactions in lung cancer.^26^, ^27^, ^28^ We studied the roles of stromal cells in the ligand–receptor crosstalk between cell compartments in the three clinical groups. Endothelial cells had the greatest strength in the recruitment of CD4+, CD8+ T cells, and macrophages through CCL14‐CCR1 signalling in the solid components (Figure 5J). Interactions related to VEGF–VEGFR crosstalk from endothelial cells to tumour cells and pericytes were more abundant in the SSNs than in the normal tissues (Figure 5K). Interestingly, stronger communication, which was related to chemo‐attraction (CCL2‐CCR2, CCL2‐ACKR1, CCL11‐ACKR1) and angiogenesis promotion (VEGF‐VEGFR), from pericytes to endothelial cells and tumour cells was identified in the solid components than in the GG components (Figure 5L). We speculated that pericytes may have distinct roles in the different radiological phenotypes of SSNs.

## 3 DISCUSSION

Early‐stage LUAD that radiologically manifests as PSNs, consisting of both GG and solid components, has distinctive growth patterns and prognosis. The images on high‐resolution CT (HRCT) scans can be suggestive of pathologic diagnoses, the GG components within PSNs tend to correlate with the histologic lepidic growth pattern, whereas the solid components correlate with invasive adenocarcinoma patterns. As described in the IASLC *8th Edition of the TNM Classification Proposals for Assessment of Tumour Size in Part‐solid Tumours*,^4^ only the long‐axis dimension of the solid components is used for staging purposes. This means that the GG components are not calculated in the tumour size for clinical T staging. Whether the biological behaviour of GG components in part solid LUAD is similar to that of nLung tissues or solid components, is a very important question. Whether the TME of GG components is similar to that of nLung tissues or solid components, and whether the TME undergoes a stepwise transition from the GG to solid components of PSNs are still unknown. In this study, we depicted the cellular landscape of the GG and solid components of PSNs, revealing their tumour cell characteristics and associated microenvironments. We also identified differences in the cell‐type composition and molecular signature expression of the different radiological appearances.

Regarding tumour cells, the GG and solid components had comparable proportions of malignant cells and shared similar CNV patterns, suggesting that the different radiological appearances were neither determined by the degree of normal‐to‐malignant transition nor associated with genomic features. This finding was consistent with our previous results.^29^ Moreover, the functional meta‐programs were identical in the two components. Interestingly, the cell‐cycle module, which is often enriched in some aggressive cancers,^28^, ^30^, ^31^ was not identified in either component, thereby revealing the underlying molecular mechanism of the indolent clinical behaviour of SSNs. In addition, our previous study showed that mutations in the gene encoding the splicing factor RNA‐binding motif protein 10 (*RBM10*) were the hallmark of SSN tumorigenesis.^29^ Another study reported that *RBM10* mutations in LUAD generally lead to the loss of function and cause extensive alterations.^32^ Consistent with these results, we found that the RNA splicing program was enriched in both components, suggesting that the high expression of genes related to RNA splicing contributes to tumour growth in GG and solid components. Signatures with the functions of stress response, immune activation, extracellular matrix remodelling/EMT, and translational initiation were identified, suggesting that the cellular states of the malignant cells in SSNs displayed the normal‐to‐malignant transition and responded to the pressure of the TME. Of note, transcriptional differences at the signalling pathway level were identified between the GG and solid components. We found that pathways related to tumour cell proliferation, invasion, and aggression were significantly up‐regulated in the solid components, which may correspond to the more aggressive behaviour of the solid components. Angiogenesis^33^, ^34^ and EMT^35^ are assumed to be the pivotal mechanisms for tumour cell invasion and aggression. The p53 signalling pathway, which is associated with apoptosis, DNA repair, and cell cycle arrest, is normally “turned off” and can be activated when cells are stressed.^36^ Its activation can be triggered by DNA damage and aberrant growth signals. We found that the p53 pathway exhibited a higher expression level in the solid component group than in the GG component group, with a higher degree of response to greater stress status. Moreover, *KRAS* mutations are driver events in LUAD and can cause abnormal activation of the KRAS pathway, through which cell proliferation, survival, and differentiation are regulated.^6^ The KRAS pathway was significantly up‐regulated in the solid components compared to in the GG components, suggesting that malignant cells in solid components have an advantage of becoming more proliferative and invasive. As a result, the cell‐cycle signalling pathway was up‐regulated in the solid components. We concluded that tumour cells in the solid components of SSNs had higher proliferation and invasiveness signatures, supporting the notion that solid components have more aggressive clinical behaviour and deserve more attention in clinical practice.

On the other hand, the TME plays a crucial role in shaping the radiological features and biological behaviour of different components within PSNs. However, whether the TME of GG components is similar to that of nLung tissues or solid components, and whether the TME undergoes a stepwise transition from the GG to solid components of PSNs are unknown. Our findings demonstrated that the relative abundance of myeloid and NK cells was highest in the nLung tissues and tended to decline stepwise from nLung tissues to the GG and solid component groups. Regarding cell subtypes, a similar distribution of immune and stromal cells was shown in the GG and solid components, but it was significantly different from that in the nLung tissues. For example, the relative percentages of CD4+, Treg, and DC subtypes in the GG components were comparable to those in the solid components but were significantly higher than those in the nLung tissues, suggesting a similar immune status in the two radiological components. These results suggested that the TME of GG components is significantly different from that of normal tissues and is similar to that of solid components.

Meanwhile, slight differences in cellular composition were seen between the GG and solid components. The percentage of CD16^high^GZMB^high^ NK cells with the strongest cytotoxic function was significantly decreased in the solid components compared to in the GG components. ACKR1+ tumour endothelial cells, which up‐regulate genes related to inflammatory response regulation, were significantly enriched in the solid components. Although only a few significant differences in the percentages of subtypes were found between the two radiological components, the functional states were already significantly different. For example, the cytotoxic scores of CD8+ effector memory T cells, GZMK+CD8+ effector T cells, and the NK subtypes were significantly lower in the solid components than those in the GG components, demonstrating a weakened cytotoxic function in the more aggressive components. Regarding the macrophages, the M1 signature expression scores were significantly higher in the GG components than in the solid components, suggesting that the pro‐inflammatory function was more powerful in the GG components than in the solid components. We also found that pericytes in the solid components had stronger communication related to angiogenesis promotion with endothelial cells and tumour cells, than those in the GG components. These results highlighted the idea that TME cells in solid components tend to show a stronger pro‐tumour function than those in GG components.

In summary, our findings offer new insight into intratumoural heterogeneity and the evolution of LUAD radiologically presenting as PSNs.

## 4 MATERIALS AND METHODS

### 4.1 Patients and sample collection

Frozen pathologically confirmed LUAD samples from 12 PSNs along with 5 paired normal controls from 12 treatment‐naive Chinese patients were subjected to single‐cell sequencing. The study design is summarised in Figure 1A, and the detailed clinical features of the cohort are summarised in Table S1. Two regions (GG and solid) from each of the 12 large PSN samples were subjected to single‐cell sequencing by 10*x* Genomics according to the gross appearance of the resected tumour and the radiological characteristics (Figure S1a, Table S1). The 5 paired normal controls were acquired from the same 12 patients. Normal control was obtained > 2 cm from the tumour edge, and judged by the frozen pathology to confirm that no cancer was examined. Pathological diagnoses were made according to the 2015 World Health Organization classification system. This study was approved by the Peking University People's Hospital Ethics Committee (2020PHB363‐01).

### 4.2 Preparation of single‐cell suspensions

All tissue samples were transported in ice‐cold H1640 (Gibco, Life Technologies) immediately after surgical resection. Then, they were rinsed with phosphate‐buffered saline (Thermo Fisher Scientific), minced into approximately 1‐mm cubic pieces, and ground with a UTTD disperser (ULTRA‐TURRAX® Tube Drive; IKA, Germany). Next, the samples were digested by 0.25% trypsin (Gibco, Life Technologies), terminated by H1640 supplemented with 2% foetal bovine serum (Gibco, Life Technologies), and then transferred to 10 ml of digestion medium containing collagenase IV (100 U/ml; Gibco, Life Technologies) and dispase (0.6 U/ml; Gibco, Life Technologies). The digested samples were filtered through a 70‐μm nylon mesh. After centrifuging, the pelleted cells were suspended with ice‐cold red blood cell lysis buffer (Solarbio) and filtered with a 40‐μm nylon mesh. Finally, the pelleted cells were suspended with 1 ml of Dulbecco's phosphate‐buffered saline (Solarbio), and the concentrations of live cells and clumped cells were determined using an automated cell counter (Countstar).

### 4.3 Droplet‐based single‐cell sequencing

Using the single‐cell 3′ Library and Gel Bead Kit V3.1 (10*x* Genomics, 1000121) and the Chromium Single Cell G Chip Kit (10*x* Genomics, 1000120), the cell suspension was loaded onto the Chromium Single Cell Controller (10*x* Genomics) to generate single‐cell gel beads in the emulsion according to the manufacturer's protocol. In short, single cells were suspended in phosphate‐buffered saline containing 0.04% bovine serum albumin. About 6000 cells were added to each channel, and the target number of cells to be recovered was estimated to be about 3000 cells. Captured cells were lysed, and the released RNA was barcoded through reverse transcription in individual GEMs. Reverse transcription was performed on an S1000TM Touch Thermal Cycler (Bio Rad) at 53°C for 45 min, followed by 85°C for 5 min, and then held at 4°C. The cDNA was generated and then amplified, and quality was assessed using an Agilent 4200 system (performed by CapitalBio Technology, Beijing, China). According to the manufacture's introduction, scRNA‐seq libraries were constructed using the Single Cell 3′ Library and Gel Bead Kit V3.1. Finally, the libraries were sequenced using an Illumina Novaseq6000 sequencer with a sequencing depth of at least 100,000 reads per cell with the paired‐end 150 bp strategy (performed by CapitalBio Technology, Beijing, China).

### 4.4 Multiplex immunohistochemistry

Formalin‐fixed/paraffin‐embedded samples from the patients included in this study were collected from Peking University People's Hospital. All the samples were cut into sections of 4‐μm thickness. The slides were deparaffinized in xylene for 30 min and rehydrated in absolute ethyl alcohol for 5 min (twice), 95% ethyl alcohol for 5 min, 75% ethyl alcohol for 2 min sequentially. Washed the slides with distilled water three times. A microwave‐oven is used for heat‐induced epitope retrieval, and during epitope retrieval the slides were I immersed in boiling EDTA buffer (pH 9.0; ZLI‐9069; Zsbio, Beijing, China) for 15 min. Antibody Diluent/Block (72424205; Perkin‐Elmer, MA, USA) was used for blocking. The antibodies used were anti‐CD4 (abcam, ab133616), anti‐CD8 (abcam, ab237709), anti‐CD19 (abcam, ab134114), anti‐CD57 (abcam, ab220187), anti‐CD11b (abcam, ab52478), anti‐CD31 (abcam, ab76533), anti‐pan cytokeratin antibody (abcam, ab32570) and anti‐PDGFR alpha + PDGFR beta (abcam, ab32570). The antigenic binding sites were visualized using the Opal 7‐Color Manual IHC Kit (Perkin‐Elmer, NEL811001KT) according to the manufacturer's protocol. Multicolour immunohistochemistry data were collected using a Mantra Quantitative Pathology Workstation (Perkin‐Elmer, CLS140089) and analysed by InForm (version 2.2.1).

### 4.5 Single‐cell RNA sequencing data pre‐processing

First, Cell Ranger v3.1.0 (10*x* Genomics) was used to demultiplex the cellular barcodes and align the reads to the human transcriptome (human reference version GRCh38) for each sample. Second, each output, which was a raw unique molecular identifier (UMI) count matrix, was transformed into a Seurat object using the R package Seurat v3.1.5.^37^ We filtered out genes that expressed in less than five cells by using the CreateSeuratObject() function with the parameter min.cells = 5. Then, several criteria were applied to each dataset to remove cells of low quality: (1) cells with fewer than 200 genes or more than 6000 genes detected, (2) cells with more than 15% of UMIs derived from mitochondrial genes, and (3) cells with more than 60,000 UMIs detected. Third, each filtered gene expression matrix was normalized and log‐transformed by the Seurat's NormalizeData() function, in which the raw gene counts for each cell were divided by the total counts for that cell, multiplied by the scale.factor (10000), and then transformed to the log‐scale (In(UMI‐per‐10000+1)). Finally, identification of 2000 variable genes of each Seurat object was performed by running Seurat's FindVariableFeatures function (*x*, selection.method = “vst,” nfeatures = 2000).

### 4.6 Multiple dataset integration

To remove batch effects and perform integrated analysis, anchors between 29 datasets were identified by using the FindIntegrationAnchors() function, and then they were passed to the IntegrateData() function. One Seurat object with a batch‐corrected expression matrix was obtained. It contained two assays: the integrated assay with the integrated expression matrix and the RNA assay with the original uncorrected value matrix. The details of the integration methods are described at https://satijalab.org/seurat/articles/integration_introduction.html.^38^ The new integrated Seurat object was used for downstream analysis.

### 4.7 Dimension reduction and identification of major cell clusters

To scale and centre the genes of the dataset, Seurat's ScaleData() function was used on the integrated slot. To reduce the dimension of the integrated object, the RunPCA() function was used with default parameters. A subset of significant PCs was selected according to the results of the ElbowPlot(), DimHeatmap() and JackStrawPlot() functions. Next, cell clustering was performed using the FindNeighbors() and FindClusters() functions. To run the uniform manifold approximation and projection (UMAP) dimensional reduction for visualization, the RunUMAP() function was implemented. Cells with common features were clustered together in the two‐dimensional UMAP map. The annotations of the major cell types (T cells, myeloid cells, epithelial cells, NK cells, endothelial cells, fibroblasts, pericytes, B cells, plasma cells, mucosa‐associated lymphoid tissue‐derived B cells and mast cells) were defined by the expression of canonical marker genes (Table S2). Doublet cells were identified by the expression of two or more canonical cell type markers and were excluded from further analyses.

### 4.8 Identification of functional cellular subsets within the major cell clusters

For the major cell types, including T cells, NK cells, myeloid cells, B cells, mucosa‐associated lymphoid tissue‐derived B cells, plasma cells, endothelial cells, epithelial cells from normal samples, fibroblasts and pericytes, were extracted and further analysed. Based on the integrated assay, the ScaleData() and RunPCA() functions were applied. The pipelines of significant PC selection, cell clustering and UMAP visualization were the same as those described above. Differentially expressed genes and specific marker genes for each cellular subset were identified using Seurat's FindAllMarkers() function with the parameter “test.use = wilcox” by default under the RNA assay. We defined each cell sub‐cluster based on the expression of canonical markers (Table S2).

### 4.9 Differentially expressed pathway analysis between different clinical groups

To characterise the significant biological processes specific to a cell type between two clinical groups, the gene set variation analysis feature of the GSVA package (version 1.34.0) was used to perform gene set enrichment analysis.^39^ The gene sets included hallmark gene sets, Kyoto Encyclopedia of Genes and Genomes (KEGG) pathways, Gene Ontology (GO) biological process terms, and REACTOME gene sets (MSigDB; http://www.broadinstitute.org/gsea/msigdb). GSVA's gsva() function was implemented to estimate the pathway enrichment scores of individual cells. The differential activities of pathways between clinical groups were calculated using the Limma R package (version 3.42.2).^40^ Significantly enriched pathways were identified using the Benjamini–Hochberg‐corrected *p*‐value of ≤ 0.01. These pathways were selected for the functional state analyses that test between clinical groups.

### 4.10 Gene signature score evaluation

To evaluate the activities of functional expression programs in one specific group (clinical group or subtype), we calculated the gene signature expression scores using Seurat's AddModuleScore() function. Gene sets of interest were first predesigned based on well‐defined markers or gene set variation analysis results. Next, the mean normalized expression value of the genes within each set was calculated for each cell. Then, the output values of the gene signatures were corrected by subtracting the mean expression value of a set of background genes. Each background gene set was randomly selected and consisted of 100 genes with expression levels that matched those of the considered gene set using 25 expression bins. Finally, for each analysis that tests between clinical groups across functional states, we fitted a function_score ∼ clinical_group + (1|patient) model with the restricted maximum likelihood (REML = TRUE) by using the lme4 and lmerTest R packages. *P*‐values were calculated using the Satterthwaite's method. The significant results were identified when the *P*‐value of the fixed effect (clinical group) in each model was reported to be < 0.05.

### 4.11 Copy number variation estimation and identification of malignant cells

Because the epithelial cells of the tumour samples comprised both malignant cells and normal cells, we identified the malignant cells by estimating their CNV levels using the inferCNV R package (https://github.com/broadinstitute/inferCNV).^41^ We chose normal epithelial cells, T cells, endothelial cells and fibroblasts as controls to define a CNV baseline. The CNV scores were calculated as the mean square of the re‐standardized CNV signals. From the epithelial cells of both the GG and solid component samples from a single patient, we extracted the cells with a sum of CNV scores in the top 5% and calculated their average CNV scores as a reference vector. Each malignant cell was identified with the CNV pattern having the Pearson correlation coefficient between its CNV pattern and the CNV reference vector above 0.3. The identified malignant cells were used for further tumour cell analysis.

### 4.12 Characterization of intratumoural expression programs

To identify the underlying intratumoural expression programs of the GG and solid components of the SSNs, the CNV‐inferred malignant cells from each sample were first normalized and centre‐scaled for each gene. Negative values in the gene expression matrix of each sample were transformed to zero. Next, the non‐negative matrix factorization was applied to each matrix using the non‐negative matrix factorization R package (version 0.23.0).^42^ To identify the robust programs of each sample, we chose the analysis pipeline as previously described.^12^ The number of factor rank (*k*) of the non‐negative matrix factorization's nmf() function ranged from 6 to 9. The initially defined expression programs were the top 50 genes ranked by non‐negative matrix factorization score for each *k*. The robust expression programs of each sample were identified as those with an overlap of at least 70% (35 of 50 genes) with a program using a different *k* value. The programs from the GG and solid samples were clustered separately. The number of overlapping genes (among the 50 top‐scoring genes of each program) was used as a similarity metric. The meta‐programs were defined by manual inspection of the hierarchical clustering results. The core genes for each meta‐program were defined as those with a minimum overlap of 25% with a program observed in another sample. The functional enrichment of the core genes was annotated using GO terms.

### 4.13 Cell–cell communication analysis

Cell–cell interactions across distinct cell types were inferred based on the expression of known ligand–receptor pairs using the CellChat software (version 1.1.1).^43^ We used the normalized data of labelled cell types as input for CellChat's createCellChat() function. Then, the pre‐processing functions, including identifyOverExpressedGenes(), identifyOverExpressedInteractions(), and projectData(), were applied with default parameters. We computed the communication probability and inferred the cellular communication network using the computeCommunProb() and filterCommunication() functions.

### 4.14 Statistics

The statistical tools, methods, and thresholds for each analysis are explicitly described with the results or are detailed in the figure legends or in Section 4.

## CONFLICT OF INTEREST

The authors declare that there is no conflict of interest that could be perceived as prejudicing the impartiality of the research reported.

## Supporting information

SUPPORTING INFORMATION
