# Supplementary material for: Single‐cell RNA sequencing reveals the multi‐cellular ecosystem in different radiological components of pulmonary part‐solid nodules
Source: Clin Transl Med. 2022 Feb 20;12(2):e723. doi: 10.1002/ctm2.723 (PMC8858630; doi:10.1002/ctm2.723)
Supplement: Supplementary file 1 — SUPPORTING INFORMATION [file CTM2-12-e723-s001.docx]

# Supplementary Material

**Supplementary Table S1. Detailed clinical information for 12 patients**

| **Individual_ID** | **Gender** | **Age** | **Smoking_history** | **Surgery_date** | **Tumor_location** | **Surgical_type** | **Pathological_type** | **Predominant**  **_subtype** | **Tumor**  **_diameter, cm** | **pTNM** | **Stage** |
| --- | --- | --- | --- | --- | --- | --- | --- | --- | --- | --- | --- |
| P3:RMYY-SSNSC-01 | female | 45 | Non-smoker | 2020/8/12 | LUL | Wedge resection | Adenocarcinoma | Acinar | 1.6 | T1bN0M0 | IA |
| P1:RMYY-SSNSC-02 | female | 62 | Non-smoker | 2020/1/7 | LUL | Segmentectomy | Adenocarcinoma | Lepidic | 1.7 | T1bN0M0 | IA |
| P4:RMYY-SSNSC-03 | male | 58 | Smoker | 2020/12/16 | RUL | Lobectomy | Adenocarcinoma | Acinar | 3.5 | T2aN0M0 | IB |
| P6:RMYY-SSNSC-04 | male | 64 | Smoker | 2021/1/29 | RUL | Lobectomy | Adenocarcinoma | Papillary | 6.8 | T3N0M0 | IIB |
| P7:RMYY-SSNSC-05 | female | 66 | Non-smoker | 2021/2/1 | LLL | Segmentectomy | Adenocarcinoma | Acinar | 2.2 | T1cN0M0 | IA |
| P8:RMYY-SSNSC-06 | female | 67 | Non-smoker | 2021/2/22 | RML | Lobectomy | Adenocarcinoma | Acinar | 2 | T1bN0M0 | IA |
| P9:RMYY-SSNSC-07 | male | 56 | Smoker | 2021/2/26 | RUL | Lobectomy | Adenocarcinoma | Lepidic | 2.8 | T1cN0M0 | IA |
| P10:RMYY-SSNSC-08 | male | 56 | Smoker | 2021/3/9 | LUL | Tri-segmentectomy | Adenocarcinoma | Acinar | 2.4 | T1cN0M0 | IA |
| P11:RMYY-SSNSC-09 | female | 48 | Non-smoker | 2021/3/15 | LUL | Lobectomy | Adenocarcinoma | Acinar | 4.3 | T2aN0M0 | IB |
| P12:RMYY-SSNSC-10 | male | 68 | Ex-smoker | 2021/3/16 | RLL | Lobectomy | Adenocarcinoma | Acinar | 2.6 | T1cN0M0 | IA |
| P2:RMYY-SSNSC-11 | female | 58 | Non-smoker | 2021/1/19 | RLL | Lobectomy | Adenocarcinoma | Acinar | 1.5 | T1bN0M0 | IA |
| P5:RMYY-SSNSC-12 | female | 37 | Non-smoker | 2020/10/26 | RLL | Lobectomy | Adenocarcinoma | Lepidic | 2.3 | T1cN0M0 | IA |
| Note: LUL,left upper lobe; LLL, left lower lobe; RUL,right upper lobe; RML, right middle lobe; RLL,right lower lobe | | | | | | | | | | | |

**Supplementary Table S2. Canonical marker genes for major cell types and subtypes.**

| Major cell type | Marker gene | Subtype | Subtype marker gene |
| --- | --- | --- | --- |
| T | *CD3D, CD3E,*  *CD3G, TRBC2,*  *TRAC, CD2* | CD4_T_Naive | *CD4, SELL, CCR7, LEF1, TCF7* |
|  |  | CD4_T_Effector_GZMA | *CD4, CD40LG, GZMA, GZMK* |
|  |  | CD4_T_Effector_HSPA1B | *CD4, CD40LG, HSPA1A, HSPA1B, IL2, TNF* |
|  |  | CD4_T_Effector_memory | *CD4, CCR6, LTB, GPR183, CCL20, IL2* |
|  |  | CD4_T_Memory | *CD4, CCR6, LTB, GPR183, ANXA1, S100A4* |
|  |  | CD4_T_Exhausted | *CD4, CXCL13, ICA1, TNFRSF4* |
|  |  | Resting_Treg | *CD4, FOXP3^lo^, IL2RA^lo^, IKZF2^hi^, CTLA4, TIGIT* |
|  |  | Suppressive_Treg | *CD4, FOXP3^hi^, IL2RA^hi^, IKZF2^hi^, CTLA4, TIGIT* |
|  |  | CD8_T**_**Effector_memory | *CD8A, ZNF683, CXCR3, IL7R, IL2, CCL5* |
|  |  | CD8_T_Effector_GZMK | *CD8A, GZMK, EOMES, KLRG1 (terminal marker)* |
|  |  | CD8_T_Naive | *CD8A, SELL, CCR7, LEF1, TCF7* |
|  |  | CD8_T_Ehausted | *CD8A, CXCL13, TIGIT, HAVCR2, LAG3* |
|  |  | MAIT | *SLC4A10* |
| NK | *FGFBP2, KLRF1,*  *KLRD1, KLRB1,*  *NKG7* | NK_FCGR3Ahi_GZMBhi | *FCGR3A^hi^, GZMB^hi^, PRF1^hi^* |
|  |  | NK_FCGR3Alo_GZMHhi | *FCGR3A^lo^*, *GZMH^hi^* |
|  |  | NK_FCGR3Alo_XCL1hi | *FCGR3A^lo^ ,XCL1, XCL2* |
| Myeloid | *LYZ, AIF1,*  *CD68, MS4A7* | Alveolar_resident_Macro_IL1Blo | *PPARG, FABP4, MARCO, IL1B^lo^* |
|  |  | Alveolar_resident_Macro_IL1Bhi | *PPARG, FABP4, MARCO, IL1B^hi^, CXCL3, CXCL20, TNF* |
|  |  | Perivascular_resident_Macro | *LYVE1, LILRB5, SELENOP**, FOLR2, SLC40A1* |
|  |  | Proliferating_Macro | *STMN1,CENPF, MKI67, TUBB, TUBA1B* |
|  |  | Classical_monocyte | *FCN1, CD14, VCAN* |
|  |  | Nonclassical_monocyte | *FCN1, FCGR3A(CD16), CDKN1C* |
|  |  | Monocyte_Derived_DC | *FCGR2B, CCL17, CLEC10A, CD1C* |
|  |  | cDC_type2 | *CD1A, CD207, HLA-DQB2, CD1C, FCER1A, FCGBP* |
|  |  | Migratory_cDC | *CCR7, LAMP3, CCL22* |
|  |  | Neutrophil_IL1Bhi | *G0S2, S100A8, S100A9, IL1B^hi^, CXCL8^hi^, SOD2* |
|  |  | Neutrophil_IL1Blo | *G0S2, S100A8, S100A9, IL1B^lo^, CXCL8^lo^* |
| Mast | *TPSAB1,TPSB2,MS4A2,CPA3* | | |
| B | *CD79A,CD79B,*  *MS4A1,BANK1* | Follicular_B_memory | *MS4A1, LTB, GPR183, BANK1* |
|  |  | Follicular_B_naive | *MS4A1, FCER2, ZBTB16* |
| Plasma and MALTB | *MZB1,JCHAIN,*  *IGHA1,IGLC2* | Plasma_IgG_immature | *IGHG3, MZB1, IGHG1, IGHG4* |
|  |  | Plasma_IgG_mature | *IGHG3, MZB1, IGHG1, IGHG2, IGHGP, XBP1* |
|  |  | MALT_B | *IGHA1, JCHAIN, IGHA2, IGHM, MZB1* |
| Endothelial | *CLDN5,VWF,*  *RAMP2,GNG11* | Tumor_EC_INSR | *INSR^hi^, HSPG2, PLVAP, VWA1, COL4A1* |
|  |  | Tumor_EC_ACKR1 | *ACKR1, INSR^lo^, SELP, IL1R1, HSPG2, VWA1* |
|  |  | Extra_alveolar_capillary_EC | *FCN3, EDN1^hi^, SLC6A4, CD36, IL7R* |
|  |  | Alveolar_capillary_EC | *HPGD, EDNRB, IL1RL1, B3GALNT1* |
|  |  | Arterial_EC | *GJA5, FBLN5, DKK2, IGFBP3* |
|  |  | Lymphatic_EC | *CCL21, TFF3, FABP4, NRP2, PDPN* |
| Fibroblast and Pericyte | *DCN,COL1A2,*  *COL1A1,ACTA2* | COL14A1_matrix_FB | *COL14A1, GSN, PI16, CFD* |
|  |  | COL13A1_matrix_FB | *COL13A1, GPC3, NPNT* |
|  |  | Myofibroblast | *ACTA2, MYH11, TAGLN, ACTG2, MYL9* |
|  |  | Pericytes | *RGS5, NOTCH3, HIGD1B, COX4I2* |
| Normal  epithelial | *EPCAM,*  *SCGB3A2,*  *SFTPB,*  *SCGB3A1* | AT1 | *AGER, CAV1, RTKN2, EMP2* |
|  |  | AT2 | *SFTPA1, SFTPA2, SFTPC, SFTPB, PGC* |
|  |  | Ciliated | *C1orf194, TMEM190, CAPS, TPPP3* |
|  |  | Club | *SCGB3A1, TSPAN8, BPIFB1, SCGB1A1* |
|  |  | Basal | *KRT15, KRT17, KRT5* |

**Note: hi: high; lo: low**
